# Site-Specific and Common Prostate Cancer Metastasis Genes as Suggested by Meta-Analysis of Gene Expression Data

**DOI:** 10.3390/life11070636

**Published:** 2021-06-30

**Authors:** Ivana Samaržija

**Affiliations:** 1 Laboratory for Epigenomics, Division of Molecular Medicine, Ruđer Bošković Institute, Bijenička 54, 10000 Zagreb, Croatia; Ivana.Samarzija@irb.hr; 2 Laboratory for Cell Biology and Signalling, Division of Molecular Biology, Ruđer Bošković Institute, Bijenička 54, 10000 Zagreb, Croatia

**Keywords:** prostate cancer, bone metastasis, lymph node metastasis, liver metastasis, gene expression, meta-analysis, focal adhesion, protein filament, androgen receptor signaling

## Abstract

Anticancer therapies mainly target primary tumor growth and little attention is given to the events driving metastasis formation. Metastatic prostate cancer, in comparison to localized disease, has a much worse prognosis. In the work presented here, groups of genes that are common to prostate cancer metastatic cells from bones, lymph nodes, and liver and those that are site-specific were delineated. The purpose of the study was to dissect potential markers and targets of anticancer therapies considering the common characteristics and differences in transcriptional programs of metastatic cells from different secondary sites. To that end, a meta-analysis of gene expression data of prostate cancer datasets from the GEO database was conducted. Genes with differential expression in all metastatic sites analyzed belong to the class of filaments, focal adhesion, and androgen receptor signaling. Bone metastases undergo the largest transcriptional changes that are highly enriched for the term of the chemokine signaling pathway, while lymph node metastasis show perturbation in signaling cascades. Liver metastases change the expression of genes in a way that is reminiscent of processes that take place in the target organ. Survival analysis for the common hub genes revealed involvements in prostate cancer prognosis and suggested potential biomarkers.

## 1. Introduction

The vast majority (90%) of cancer-related deaths is not caused by primary tumors but metastasis on distal organs [1]. Still, cancer chemotherapies are mainly designed in a way that they consider events that drive primary tumor growth only, and little attention is given to pathways governing metastatic outgrowth [2]. While the literature and research on gene expression differences between primary tumors and metastasis is abundant [3], little is known on which signaling pathways are shared among metastatic cells colonizing distinct secondary sites, and which strategies are site-specific. Several publications exist on such topics, including the recent reports by Hartung et al. [4,5]. This type of knowledge is essential to suggest signaling pathways that could be therapeutic targets in metastatic cells in cancer types that spread to multiple organs. 

Prostate cancer (PCa) is the second most commonly occurring cancer in men and the fifth leading cause of death worldwide [6]. The 5-year survival rate of non-metastatic prostate cancer is 98.9%, but, the rate in patients who are initially diagnosed with metastatic prostate cancer is less than 30% and does not show improvements [7]. The most usual sites colonized by prostate cancer cells are bones, lymph nodes, lungs, liver, and brain while rare locations include adrenal glands, breasts, eyes, kidneys, muscles, pancreas, salivary glands, and spleen. Recently, it was shown that patients with liver-only metastasis have worse cancer-specific and overall survival than patients with bone-only and lung-only metastasis [8].

The driving events in prostate cancer dissemination include entangled actions of several signaling pathways that are potentiated by changes in gene expression, genetic alterations [9], and post-translational modifications [10]. To better understand signaling events that are site-specific and common to prostate cancer metastasis, herein a meta-analysis of publicly available GEO gene expression datasets that consist of primary prostate cancer samples as well as samples of metastasis from bones, lymph nodes, and liver was conducted. Differentially expressed genes that are shared among all sites analyzed are presented. A substantial number of differentially expressed genes are secondary site-specific which emphasizes the need to study metastasis separately according to the secondary site. Survival analysis for hub genes found among the genes commonly changed in all metastatic sites was conducted and has revealed potential biomarkers.

## 2. Materials and Methods

### 2.1. Gene Expression Datasets

The list and description of gene expression datasets downloaded from the GEO database [11] and used in the meta-analysis are provided in Table 1. All the datasets are from microarray chips. The samples within datasets belong to four categories: primary tumors, bone metastasis (51 samples versus 175 primary tumor samples, from three datasets), lymph node metastasis (103 samples versus 232 primary tumor samples, from four datasets), and liver metastasis (26 samples versus 83 primary tumor samples, from two datasets). Depending on the platform, the annotation was either downloaded from the file deposited on the GEO database or obtained using gProfiler [12].

### 2.2. Meta-Analysis of Gene Expression Data and Enrichment Analysis

Meta-analysis of the gene expression data was performed using ImaGEO software that displayed good performance in the comprehensive gene expression meta-analysis [18]. The *p*-value method (minP) and default settings were used. This meta-analysis method was chosen as combining *p*-values provides an advantage over effect size combination for standardization of the associations from genomic studies to a common scale allowing to compare very heterogeneous datasets, for example, datasets from different tissues [18]. The criteria for differential gene expression were false discovery rate (FDR) *p*-value < 0.05 and ǀlog2fold changeǀ > 1. The intersection and the list of genes differentially expressed among bone, lymph node, and liver metastasis were obtained using GeneVenn [19]. Overview of enrichment analysis was obtained by Enrichr [20] using default settings and GO Biological Process (BP), GO Cellular Component (CC), GO Molecular Function (MF), KEGG (Kyoto Encyclopedia of Genes and Genomes), and WikiPathways data are presented. The top five processes are listed. The background used is set by default by Enrichr software, as Enrichr cannot upload a background list [20].

### 2.3. Visualization of Metastasis Genes Networks and Identification of the Hub Genes

The networks representing up-regulated and down-regulated genes among the 434 shared metastasis genes were retrieved by STRING [21] with default settings (default medium confidence 0.4) and visualized in Cytoscape [22]. The genes that showed interactions are depicted as a network, while the genes with no connections were omitted from the Figures. Twenty hub genes from a set of 434 genes that are shared among all metastatic sites were detected with the use of cytoHubba application [23].

### 2.4. Survival and Expression Analysis for the Hub Genes

The prognostic significance of each hub gene was performed by gene expression profiling interactive analysis (GEPIA [24]) taking into account disease-free survival. *p* < 0.05 was considered to indicate a statistically significant difference.

The analysis of mRNA expression for the first 10 hub genes was performed using GEPIA software, including TCGA (The Cancer Genome Atlas) and GTEx (Genotype-Tissue Expression) data.

## 3. Results

### 3.1. The Top Ten Most Changed Genes Shared by Metastasis from All Analyzed Sites Belong to the Class of Filaments and Proteins Involved in Bone and Prostate Biology

As shown in Table 2, a substantial proportion (40 of 260) of genes that are among the 50 most up-regulated or 50 most down-regulated in prostate cancer bone, lymph node, and liver metastasis groups are shared at least between two groups (30 genes) or between all three groups (10 genes). It is interesting to note that all 40 genes that are found overlapping in two or three groups change in the same direction (up- or down-regulated), adding to the hypothesis that the change in their expression is functional, “driving” change rather than “passenger” change. The ten genes found in all groups are SPP1 (up-regulated) and MYH11, MSMB, ACTG2, CNN1, PCP4, KRT15, NEFH, DES, and CHRDL1 (down-regulated).

According to the KEGG enrichment analysis of the nine shared down-regulated genes MYH11 and ACTG2 belong to the enriched process of vascular smooth muscle contraction (*p*-value = 0.0015). Additionally, five of those nine genes are also either filaments or they are involved in their processes, as listed below. CNN1 is a thin filament-associated protein that is also implicated in the regulation and modulation of smooth muscle contraction. DES is a muscle-specific type III intermediate filament essential for proper muscular structure and function. NEFH is an intermediate filament protein, part of neurofilaments. KRT15 is a keratin that belongs to intermediate filament proteins responsible for the structural integrity of epithelial cells. PCP4 is a calmodulin regulator protein that functions as a modulator of calcium-binding by calmodulin. Among other roles, it was shown that Ca2+ and calmodulin regulate the binding of FLNA to actin filaments [25]. In summary, MYH11 and ACTG2 genes belong to genes involved in the assembly of actin fibers, while CNN1 and PCP4 are proteins capable of binding actin or influence its association with partner proteins. KRT15, NEFH, and DES belong to the three (keratins, neurofilaments, and desmin, respectively) of five classes of intermediate filaments.

SPP1 gene codes for osteopontin, the protein that is involved in the attachment of osteoclasts to the mineralized bone matrix. CHRDL1 protein has recently been shown to improve osteogenesis of bone marrow mesenchymal stem cells [26]. CNN1 gene also plays a role in osteoblast and osteoclast function and formation [27].

MSMB is one of the three major proteins secreted by the epithelial cells of the prostate. It is also secreted by epithelial cells in many other organs. The protein inhibits the growth of cancer cells in an experimental model of prostate cancer, but this property was shown to be cell line-specific [28].

### 3.2. Reorganization of Focal Adhesions and Changes in Androgen Receptor Signaling Are Common Characteristics of Prostate Cancer Metastasis Regardless of the Target Organ

**Figure 1:**
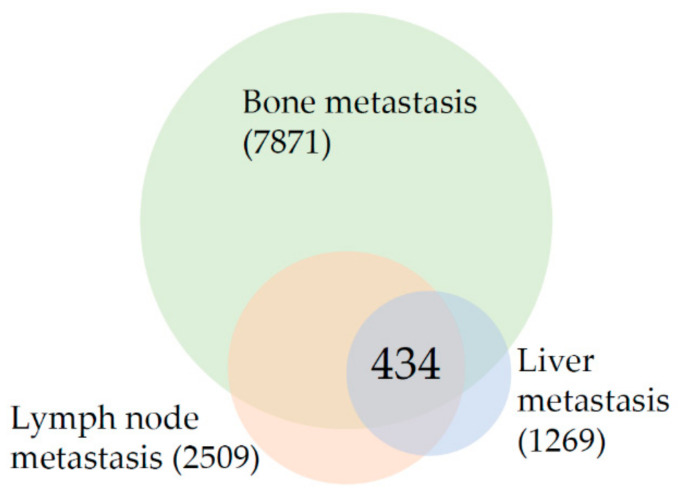
Venn diagram of differentially expressed genes in prostate cancer bone, lymph node, and liver metastasis in comparison to primary prostate tumors. The number in parenthesis indicates the total number of differentially expressed genes and the number in the intersection indicates the number of overlapping genes.

**Figure 2:**
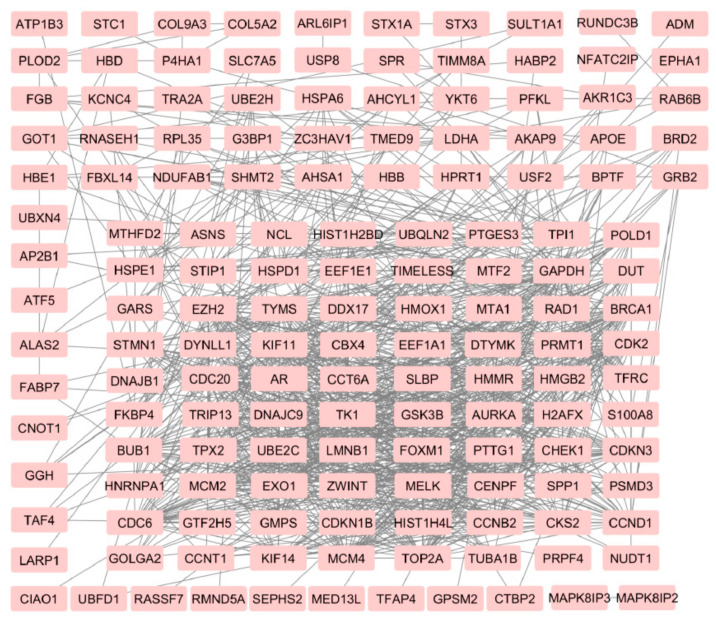
Network of up-regulated genes in prostate cancer metastasis from an intersection of bone, lymph node, and liver metastasis.

**Figure 3:**
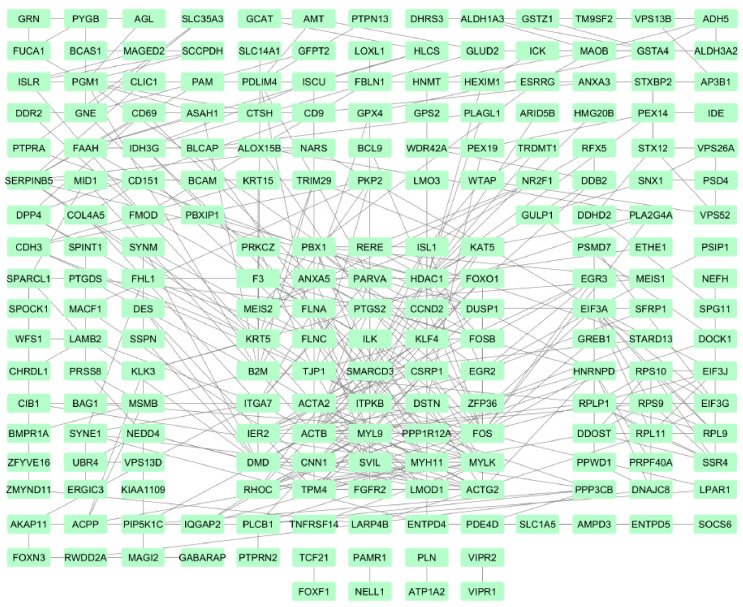
Network of down-regulated genes in prostate cancer metastasis from an intersection of bone, lymph node, and liver metastasis.

As shown in Figure 1, the meta-analysis revealed that the intersection of differentially expressed genes among prostate cancer bone, lymph node, and liver metastasis consists of 434 genes. This gene list was analyzed to establish prostate cancer metastasis “core transcriptional program”. Table 3 is listing the results of enrichment analysis for all 434 shared genes. It is clear from Table 3 that “Focal adhesion” is the most changed enrichment term in the intersection of metastasis from all sites as it is among the top enriched terms from the three lists–KEGG, WikiPathways, and GO CC. The up-regulated and down-regulated gene networks are depicted in Figure 2 and Figure 3, respectively.

The enrichment term “Prostate cancer” with 10/97 genes, “miRNA regulation of prostate cancer signaling pathways” with 8/33 genes, and “Androgen receptor” (12/90) are also among the most changed enrichment terms (Table 3) confirming the specificity of the results.

### 3.3. Results Suggest That Transcriptional Landscape of Bone Metastasis Is Profoundly Rewired in Comparison to Lymph Node and Liver Metastasis

In comparison to the number of genes differentially expressed in lymph nodes (2509) and liver (1269) metastasis, the changes in bone (7871 genes) metastasis are several times higher, suggesting the profound change in the transcriptional repertoire. Enrichment analysis was done for all the genes that are changed in bone metastasis (Table 4). On the top of the list of genes whose expression changes in bones is the term “VEGFA-VEGFR2 signaling pathway” followed by genes from focal adhesions and other signaling pathways. The BP category showed enrichment in genes involved in neutrophil biology. The top of the enrichment list of differentially expressed genes found in bone metastasis only (data not shown) is dominated by genes involved in immune system function, especially “Chemokine signaling pathway”, “T-Cell antigen receptor signaling pathway” and “B-cell receptor signaling pathway”.

The enrichment analysis of 100 most up- or down-regulated genes (Table 2) in bone metastasis revealed enriched term of “Regulators of bone mineralization” (IBSP, COL4A1, and SPP1) being on the top of the list (BioCarta 2016). 

### 3.4. Lymph Node Metastasis Are Characterized by Changes in Signaling Networks While the Liver Metastasis Transcriptional Program Is Reminiscent of Processes That Take Place in the Target Organ

On the top of the list of genes that are changed in lymph node metastasis (Table 5) is the term “VEGFA-VEGFR2 signaling pathway”, followed by “Focal adhesion”. This was found to be similar to the result of the genes differentially expressed in bone metastasis. Other signaling pathways whose components are found to have significant differential expression in lymph node metastasis include PI3K-Akt, EGF/EGFR, MAPK, and TGF-beta signaling pathways.

On the top of the list of genes that are changed in liver metastasis (Table 6) is the term “VEGFA-VEGFR2 signaling pathway”. When the enrichment analysis for the 100 most up- or down-regulated genes (Table 2) in liver metastasis was done, the term “Complement and coagulation cascades” was found to be highly enriched with 13/79 genes being present on the list (data not shown). Genes specifically changed in liver metastasis only (data not shown) are reminiscent of processes taking place in the target organ according to the terms that are enriched and that include “Folate metabolism”, “Selenium micronutrient network”, “Fat digestion and absorption”, “Cholesterol metabolism”, “Phenylalanine metabolism” and fore-mentioned “Human complement system” and “Complement and coagulation cascades”.

### 3.5. Prostate Cancer Metastasis Hub Genes and Their Involvement in Patient Disease-Free Survival

**Figure 4:**
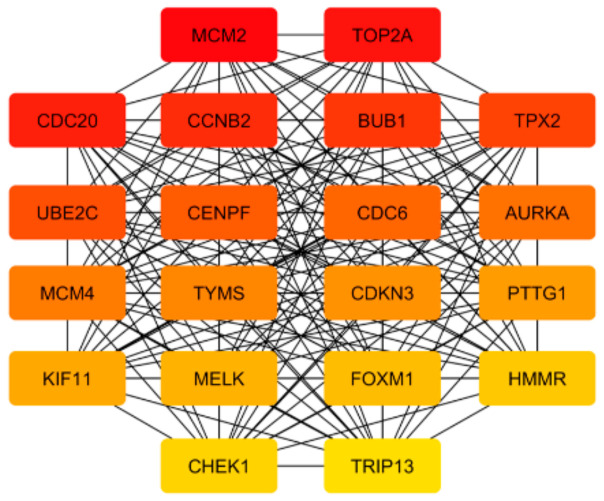
Hub genes that are found among 434 overlapping genes that are changed in metastases from all sites analyzed. The highest-ranked node is in red, and the lowest in yellow.

**Figure 5:**
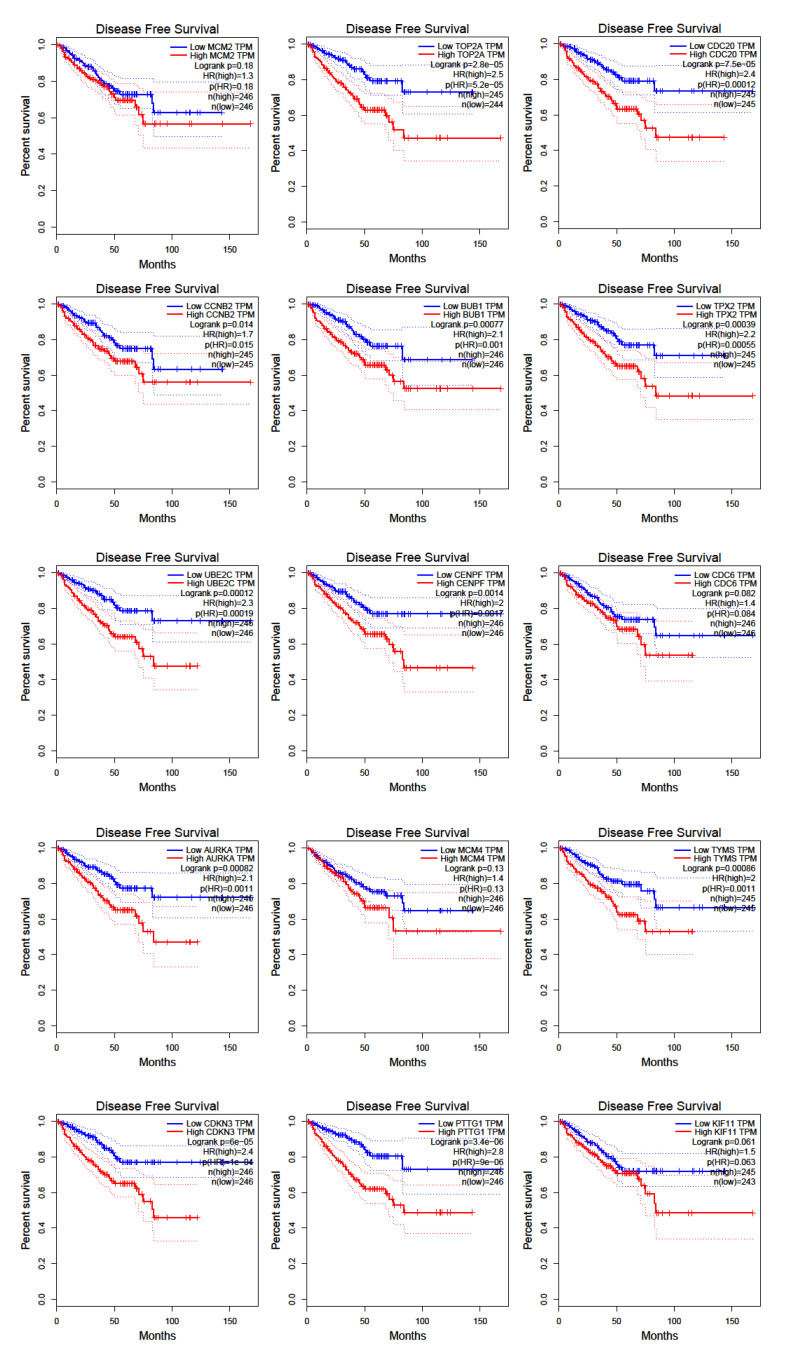

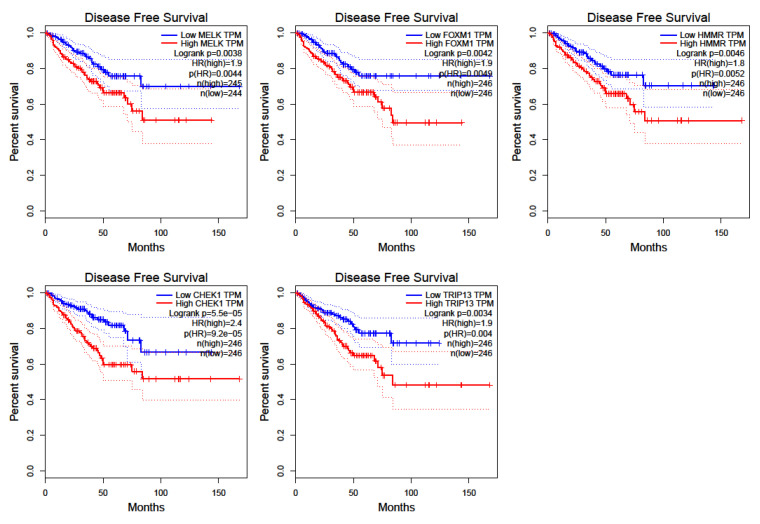
Disease-free survival for hub genes as retrieved by GEPIA software using TCGA data.

**Figure 6:**
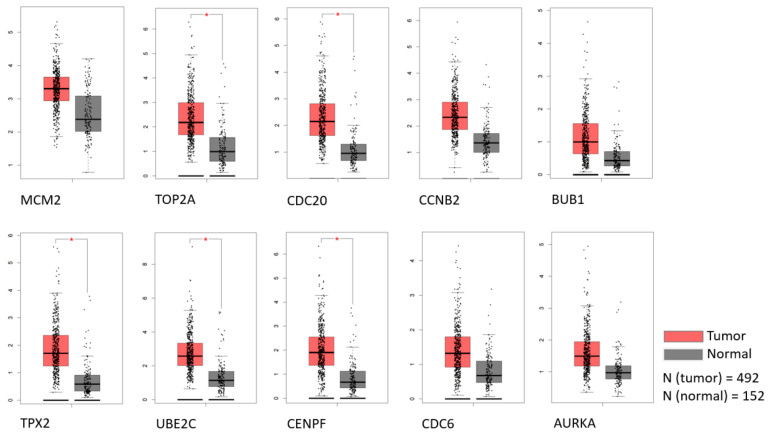
The expression of the first 10 hub genes by rank in TCGA and GTEx datasets. Statistically significant differences are marked with an *.

To detect the potential driving network of prostate cancer metastasis, hub genes among the 434 common genes of all metastatic sites were singled out and are shown in Figure 4. The disease-free survival analysis revealed the statistically significant involvement of the following genes (Figure 5): AURKA, BUB1, CCNB2, CDC20, CDKN3, CENPF, CHEK1, FOXM1, HMMR, MELK, PTTG1, TOP2A, TPX2, TRIP13, TYMS, UBE2C. Their biological roles are listed in Table 7 and among the enriched terms listed in Table 8 are “Cell-cycle” and “Microtubule cytoskeleton”. The analysis of mRNA expression for the first ten hub genes is shown in Figure 6, confirming their up-regulation in prostate cancer.

## 4. Discussion

Metastasis formation is a complex process driven by a variety of genes and molecular events [3]. Meta-analysis on differential gene expression from primary tumors and metastasis are common for different cancer types. However, the analyses that stratify metastatic samples according to the secondary sites are rare with more interest shown in very recent years [29,30,31,32,33]. To the best of my knowledge, the work that is presented here is the first such study for prostate cancer and its most common metastasis sites. These types of studies are important as they accumulate information that could be of interest when designing anti-metastatic therapies. 

The main finding of the presented study is that a group of differentially expressed genes encoding for filaments or associated proteins is the most differentially expressed by fold change and the processes of focal adhesion and androgen receptor signaling are among the most changed in metastasis from all sites analyzed. Moreover, there is a substantial difference in expression programs from metastasis from different sites. In the following chapters, based on the results, several questions are considered: what are the site-specific transcriptional programs that predispose or characterize the metastasis from bone, lymph node, and liver and could potentially be used as targets to treat metastasis from those particular sites; what are the common genes that could be used as targets to treat metastasis from all sites; what are the strategies that are used by cancer cells to colonize different organs?

Although the lymph nodes are the first sites encountered by prostate cancer cells that enter the circulation system, the bones are the most common sites that are homing them [34]. This is very intriguing as this study shows that, among the three most common distal sites, metastasis found in bones underwent a much more profound change of transcriptional program (7871 genes with changed expression) in comparison to metastasis from lymph nodes (2509) and liver (1269). The question arises as to what facilitates the colonization of the bones when, despite the complete reorganization of the transcriptional program that is needed, they are still the first choice for prostate cancer cell homing? The suggested concept of pre-metastatic niche offers the explanation that the primary cancer cells, possibly through exosomes, prime the distal organs which increases chances and enables their homing [35]. However, the question is whether there is a predisposition already within primary prostate cancer cells and their transcriptional program that is kept and subsequently upgraded in metastatic cells that makes them prone to bone colonization (“seed pre-selection” [36]). From a list of differentially expressed genes, the driving events in bone-specific metastases that could be directing them to the target organ are extracted. Upregulation of SPP1 and downregulation of CHRDL1 and CNN1 (genes involved in bone biology) in metastasis from all sites analyzed are found which suggests they are changed early on and could belong to the genes that make prostate cancer metastatic cells bone-gravitating. The expression level of SPP1 is elevated in cancers, particularly those that spread preferentially to the skeleton. “Osteomimicry” of malignant cells is partially conferred by transmembrane receptors bound by SPP1. Binding of integrins on malignant cells by SPP1 results in activation of signaling cascades within the cell that promotes metastasis [37]. CHRDL1 gene encodes an antagonist of bone morphogenetic protein 4 and may play a role in embryonic bone formation, while overexpression of CNN1 in osteoblast lineage cells was shown to regulate bone mass [27]. Because of their prominent role in bone biology as listed above, these genes could contribute to the site-preference during the metastatic process. Also, an extensive change in transcription of immune cell-related genes (chemokines, T- and B-cells, and neutrophil-related genes) was recorded in genes differentially expressed in bone metastasis. This transformation supports the role of prostate cancer cell and immune system crosstalk which is crucial for the formation of metastasis in this organ [38]. The contribution of chemokines to the metastatic process has been well documented [39,40]. For example, the prostate cancer metastasis-promoting role of CXCL5 has been recently shown [41]. Some other chemokines from the list of differentially expressed genes (e.g CCL5, CCR2, reviewed in [40]) are also implicated in the metastatic process from different cancer types. On the list of differentially expressed chemokines in bone metastasis (data not shown), 13 out of 14 chemokines or chemokine receptors with changed expression in bone metastasis only, are up-regulated, indicating a strong increase of activity in the network of chemokines with known (minor part) and yet to be investigated (major part) roles in prostate cancer metastasis. This finding suggests that targeting the chemokine signaling pathway could alleviate the bone metastasis burden in prostate cancer patients.

As noted above, to colonize lymph nodes and liver, prostate cancer cells undergo changes in transcription that are several times less extensive in the number of affected genes than in bone metastasis. From this data, it could be suggested that the brute force that drives prostate cancer cells through circulation is probably more involved in regional lymph node and liver colonization than in bones. However, enrichment analysis of genes whose expression is changed in lymph nodes only revealed extensive changes in the expression of kinases, suggesting rewiring of signaling cascades. While the term “Focal adhesion” was on the top of the enrichment lists of genes shared among all metastasis, this list was extended to 60 genes, including 7 integrins, and was on the top of the list shared among bone and lymph node but not liver metastasis (data not shown). This indicates that bone and lymph node metastasis highly rewire the expression of adhesion molecules and related pathways, while for liver metastasis this change is much less prominent. Taken together, these results suggest that there are parts of focal adhesion (integrin) network that are commonly changed in prostate cancer metastasis from all analyzed sites, but also a part that is specific to metastasis from each site, giving them an integrin code that is, apparently, important during every step of the metastatic cascade [42] and a subject to a frequent change [43].

The observation from this work which indicates that the expression of genes encoding for microfilaments and intermediate filaments or associated proteins (MYH11, ACTG2, KRT15, NEFH, DES, CNN1, and PCP4) are the most extensively down-regulated in prostate cancer metastasis from all sites suggests fundamental reorganization of their cytoskeleton. KRT15 is downregulated in the progression of normal prostate tissue to prostate cancer and further to lymph node metastasis [44]. Other mentioned genes influence tumors either by inhibiting [45,46] or promoting their pathogenicity [47,48] or displaying a dual role [49,50]. Those studies investigated the roles of individual proteins, however, in this system, it is very likely that they act in concert which could be envisioned from their involvement in the same process of cytoskeleton assembly.

It is interesting to note that liver metastases are highly enriched for genes that are involved in the processes of a target organ. This phenomenon of metastatic cells adapting to the host site has been recently described [5], but the potential contamination with the host tissue should also be taken into account.

The role of androgen receptor signaling in prostate cancer progression is multilayered and has been extensively studied [51,52]. The change in androgen receptor signaling pathway (up-regulation of androgen receptor and changes in expression of related genes) that are documented here are in agreement with recent findings that elevation of androgen receptor promotes prostate cancer metastasis by induction of epithelial-mesenchymal transition [53]. Also, this result is in agreement with a study by Guo et al. [54] who suggest androgen receptor as one of the hub genes in metastatic prostate cancer. However, the study presented here encompasses a larger sample size and presents a more extended approach. In addition, herein differences in differential gene expression between different secondary sites are in focus which is, to the best of my knowledge, a unique approach for prostate cancer metastasis and not so common for other cancer types. 

In this study, 20 genes were singled out as hub genes among those that change expression in all metastatic sites analyzed. For most of these genes involvement in disease-free survival was shown suggesting that these genes might be considered in potential targeted therapies.

Finally, heterogeneity of prostate cancer calls for studies that include an even larger number of samples than this study did. The experimental validation of these results would bring the confirmation of the importance of the genes that are suggested here to play a role in the prostate cancer metastatic process. Also, this analysis involved only the data for protein-coding genes and their subsequent mRNA expression. However, the roles of non-coding RNAs are also known to be highly important in prostate cancer progression, both as regulatory elements and biomarkers [55,56]. It would be interesting to reveal their roles in prostate cancer metastasis from different secondary sites.

## 5. Conclusions

Although sharing changes in the expression of basic groups of genes belonging to the class of filaments and focal adhesions and androgen signaling pathway, metastasis from different sites differ profoundly in their transcriptional program. Based on this finding, it can be concluded that it is important to study separately cancer cells originating from different secondary sites because the results of gene expression data are expected to be skewed when metastasis samples are not stratified. In addition, data on the differentially expressed genes that are site-specific and common to all metastases provide potentially useful information for targeting metastatic cells. It would be interesting to see whether metastatic cells from different primary organs use similar strategies when colonizing the same secondary site. AURKA, BUB1, CCNB2, CDC20, CDKN3, CENPF, CHEK1, FOXM1, HMMR, MELK, PTTG1, TOP2A, TPX2, TRIP13, TYMS, UBE2C are the hub genes identified in this study that show involvement in the disease-free survival of prostate cancer patients.

## Figures and Tables

**Table 1:** Description of datasets used in this study.

GEO Set	Reference	Platform	Samples			
			Primary Tumors	LN Met.	Liver Met.	Bone Met.
GSE6605, GSE6606	Chandran et al. 2007 [13]	GPL8300	61	15	5	-
GSE21034	Taylor et al. 2010 [14]	GPL10264	131	7	-	2
GSE32269	Cai et al. 2013 [15]	GPL96	22	-	-	29
GSE59745	Böttcher et al. 2015 [16]	GPL5175	18	12	-	-
GSE77930	Kumar et al. 2016 [17]	GPL15659	22	69	21	20

**Table 2:** 50 most up- (first row) and down-regulated (second row) genes in prostate cancer metastasis originating from bones, lymph nodes, and liver. The genes are ordered by decreasing ǀfold changeǀ. Genes shared among three lists are depicted in red; genes shared among bone and LN metastasis are depicted in blue; genes shared among bone and liver metastasis are depicted in orange; genes shared among LN and liver metastasis are depicted in green.

Bone Metastasis	Lymph Node Metastases	Liver Metastasis
COL11A1, SPP1, HBB, IBSP, HBD, FABP4, MMP13, TNFAIP6, COL5A2, LRRC15, S100A8, MMP9, PTX3, TOP2A, FMO3, AKR1C3, COL1A1, SLPI, CA1, COL3A1, ENPEP, GMFG, COL1A2, CD36, LPL, OMD, APOE, SERPINE2, CFH, IGF2BP2, AHSP, TPX2, OLFML2B, CYP26A1, KIF20A, MEPE, COL10A1, MKI67, CTSZ, BUB1, DMP1, DPT, COL5A1, PPBP, IFI27, SULT1B1, COL4A1, STAB1, KIF4A, ALAS2	CDKN3, THOC5, CCNE2, PPP3CA, TPH1, RFC5, UBE2C, HIST1H4L, FABP4, PAK2, PMAIP1, ESM1, MPHOSPH9, RAD23B, MVD, CENPF, SLC26A3, CD36, SPP1, RIPK2, RFPL3S, HMGCS1, TIMM8A, VDR, KCTD20, ATP8A2, NTNG1, BUB1, MAGEA12, CDC6, TOP2A, UBFD1, PTTG1, OSGIN2, COL9A3, KRR1, COL11A1, EZH2, ANGPT2, THBS2, FGF12, FOXM1, MYL4, KIF3A, CDKN2A, TPX2, NCBP1, MLF1, SEL1L, LMNB1	FGB, FGG, PCK1, GC, FABP1, CRP, HP, CFHR4, APOC3, TM4SF4, CDKN3, APOB, APOA1, FGL1, RBP4, PEG10, HPGD, UGT2B7, ARG1, PLG, F2, FGA, SERPINC1, HBD, ALDOB, ALB, AMBP, SLC1A2, HIST1H4L, SPP1, AKR1C4, KNG1, KIF11, NMU, SERPINA1, COL9A3, APCS, IGFBP1, CPS1, F9, AADAC, MKNK2, UBE2C, ASGR2, AHSG, C4BPA, KIF23, C8A, SAA1, HSPA6
ACADL, PGM3, SORD, MT1E, ANXA3, SMARCA1, KIAA1324, CHRDL1, MYBPC1, RLN1, PAK1IP1, ANPEP, GULP1, ACTA2, JMJD1C, NR4A2, LIFR, HSD17B6, DHRS7, KLF6, EPHX2, KIF5C, SFRP1, IQGAP2, MYLK, DMXL1, NCAPD3, CPE, SLC4A4, GREB1, FOS, ALDH1A3, ZFP36, HGD, DES, AZGP1, NEFH, DPP4, ABCC4, TRPM8, KRT15, FOSB, MEIS2, SORBS1, PCP4, CNN1, MAOB, ACTG2, MSMB, MYH11	LTF, FBLN1, EGR2, AKAP5, ABCA8, SLC20A2, EGR3, FHL1, KRT5, PAMR1, MYLK, BDNF, EYA4, CHRDL1, EPHB6, NR4A2, HDAC9, GPM6B, EYA1, ATP1A2, EDNRA, CXCL11, PTGS2, FGFR2, PTPRD, KRT15, IGFBP6, PLN, SPOCK3, RLN1, DKK1, MFAP4, PTN, MOXD1, MSMB, PTGDS, MMP7, MEIS1, FOXF1, SYNM, CNTN1, DES, TCF21, CNN1, PCP4, OLFM4, NEFH, PAGE4, ACTG2, MYH11	NT5E, PHF14, SPOCK3, VWF, NOV, CPM, NR4A3, COL16A1, EGR2, FGFR2, DYNC1I1, VPS13D, DDR2, PLA2G4A, TAC1, EGR3, CDH11, KRT15, FRZB, ATP1A2, SFRP1, SULF1, FOXF1, EDNRA, MYL9, PCP4, SPARCL1, UBE2K, CHRDL1, IGFBP6, DES, MFAP4, PTGDS, ACTC1, KLK11, SLC20A2, PAGE4, KLRD1, FBLN1, NEFH, MSMB, CNN1, KLK2, HDAC9, RLN2, CNTN1, PKP2, ACTG2, F3, MYH11

**Table 3:** Gene ontology and pathway enrichment analysis of the genes common to all metastatic sites analyzed.

Category	Term	Count	Adj. *p*-Value
BP	Platelet aggregation (GO:0070527)	8/33	0.0010
	Negative regulation of cellular macromolecule biosynthetic process (GO:2000113)	30/512	0.0011
	Homotypic cell-cell adhesion (GO:0034109)	8/38	0.0011
	Regulation of retinoic acid receptor signaling pathway (GO:0048385)	5/13	0.0027
	Neutrophil degranulation (GO:0043312)	27/479	0.0027
CC	Cytoskeleton (GO:0005856)	32/520	3.37 × 10^−5^
	Focal adhesion (GO:0005925)	24/356	1.31 × 10^−4^
	Ficolin-1-rich granule (GO:0101002)	15/184	9.15 × 10^−4^
	Microtubule cytoskeleton (GO:0015630)	23/388	9.15 × 10^−4^
	Chromatin (GO:0000785)	19/296	0.0014
MF	Kinase binding (GO:0019900)	30/418	5.99 × 10^−6^
	Protein kinase binding (GO:0019901)	30/495	1.22 × 10^−4^
	Cadherin binding (GO:0045296)	19/313	0.0103
	Metal ion binding (GO:0046872)	23/442	0.0143
	Kinesin binding (GO:0019894)	5/28	0.0268
KEGG	Focal adhesion	20/199	3.7 × 10^−6^
	Cell cycle	14/124	6.72 × 10^−5^
	Prostate cancer	10/97	0.0042
	Glycolysis/Gluconeogenesis	8/68	0.0071
	p53 signaling pathway	8/72	0.0085
WikiPathways	Focal Adhesion WP306	19/198	2.17 × 10^−5^
	Cell Cycle WP179	14/120	4.16 × 10^−5^
	miRNA regulation of prostate cancer signaling pathways WP3981	8/33	4.16 × 10^−5^
	Androgen receptor signaling pathway WP138	12/90	4.37 × 10^−5^
	DNA Damage Response WP707	10/68	1.24 × 10^−4^

**Table 4:** Gene ontology and pathway enrichment analysis of the genes differentially expressed in bone metastasis.

Category	Term	Count	Adj. *p*-Value
BP	Cellular protein modification process (GO:0006464)	582/1001	3.21 × 10^−31^
	Neutrophil activation involved in immune response (GO:0002283)	320/483	2.26 × 10^−30^
	Neutrophil mediated immunity (GO:0002446)	322/487	2.26 × 10^−30^
	Neutrophil degranulation (GO:0043312)	317/479	4.20 × 10^−30^
	Positive regulation of gene expression (GO:0010628)	459/771	7.62 × 10^−28^
CC	Focal adhesion (GO:0005925)	245/356	3.51 × 10^−27^
	Secretory granule lumen (GO:0034774)	217/317	1.00 × 10^−23^
	Nuclear body (GO:0016604)	350/618	1.37 × 10^−16^
	Mitochondrion (GO:0005739)	537/1026	4.30 × 10^−16^
	Nucleolus (GO:0005730)	375/676	4.63 × 10^−16^
MF	RNA binding (GO:0003723)	794/1387	2.90 × 10^−41^
	Cadherin binding (GO:0045296)	221/313	9.62 × 10^−27^
	Protein kinase activity (GO:0004672)	308/513	3.11 × 10^−19^
	Protein homodimerization activity (GO:0042803)	380/664	6.99 × 10^−19^
	Protein kinase binding (GO:0019901)	295/495	6.33 × 10^−18^
KEGG	Pathways in cancer	335/530	7.73 × 10^−27^
	Human T-cell leukemia virus 1 infection	155/219	4.57 × 10^−19^
	Human papillomavirus infection	214/330	3.89 × 10^−19^
	PI3K-Akt signaling pathway	222/354	2.39 × 10^−17^
	Protein processing in the endoplasmic reticulum	121/165	3.98 × 10^−17^
WikiPathways	VEGFA-VEGFR2 Signaling Pathway WP3888	166/236	1.57 × 10^−19^
	Focal Adhesion-PI3K-Akt-mTOR-signaling pathway WP3932	198/303	7.32 × 10^−18^
	Focal Adhesion WP306	141/198	1.17 × 10^−17^
	EGF/EGFR Signaling Pathway WP437	120/162	2.70 × 10^−17^
	TGF-beta Signaling Pathway WP366	102/132	5.84 × 10^−17^

**Table 5:** Gene ontology and pathway enrichment analysis of the genes differentially expressed in lymph node metastasis.

Category	Term	Count	Adj. *p*-Value
BP	Regulation of transcription from RNA polymerase II promoter (GO:0006357)	303/1478	9.34 × 10^−16^
	Positive regulation of transcription, DNA-templated (GO:0045893)	241/1120	8.53 × 10^−15^
	Positive regulation of transcription from RNA polymerase II promoter (GO:0045944)	195/848	8.73 × 10^−15^
	Negative regulation of apoptotic process (GO:0043066)	130/485	9.64 × 10^−15^
	Regulation of cell migration (GO:0030334)	96/316	2.69 × 10^−14^
CC	Focal adhesion (GO:0005925)	110/356	1.26 × 10^−17^
	Cytoskeleton (GO:0005856)	127/520	8.73 × 10^−12^
	Membrane raft (GO:0045121)	46/119	5.95 × 10^−11^
	Perinuclear region of cytoplasm (GO:0048471)	96/378	5.33 × 10^−10^
	Platelet alpha granule (GO:0031091)	36/90	3.67 × 10^−9^
MF	Protein kinase binding (GO:0019901)	148/49	3.44 × 10^−22^
	Kinase binding (GO:0019900)	124/418	2.94 × 10^−18^
	Cadherin binding (GO:0045296)	101/313	1.19 × 10^−17^
	RNA binding (GO:0003723)	274/1387	4.87 × 10^−13^
	RNA polymerase II regulatory region sequence-specific DNA binding (GO:0000977)	116/460	1.11 × 10^−11^
KEGG	Pathways in cancer	136/530	2.06 × 10^−14^
	Focal adhesion	70/199	2.06 × 10^−14^
	MAPK signaling pathway	86/295	1.89 × 10^−12^
	PI3K-Akt signaling pathway	94/354	4.25 × 10^−11^
	Human T-cell leukemia virus 1 infection	67/219	7.58 × 10^−11^
WikiPathways	VEGFA-VEGFR2 Signaling Pathway WP3888	80/236	5.16 × 10^−15^
	Focal Adhesion WP306	69/198	8.89 × 10^−14^
	Integrated Breast Cancer Pathway WP1984	52/151	4.22 × 10^−10^
	Cell Cycle WP179	44/120	9.84 × 10^−10^
	Ebola Virus Pathway on Host WP4217	46/129	9.84 × 10^−10^

**Table 6:** Gene ontology and pathway enrichment analysis of the genes differentially expressed in liver metastasis.

Category	Term	Count	Adj. *p*-Value
BP	Cellular protein metabolic process (GO:0044267)	76/484	7.17 × 10^−10^
	Platelet degranulation (GO:0002576)	33/124	1.90 × 10^−9^
	Regulated exocytosis (GO:0045055)	35/148	1.07 × 10^−8^
	Negative regulation of cellular process (GO:0048523)	73/534	4.23 × 10^−7^
	DNA metabolic process (GO:0006259)	51/314	4.31 × 10^−7^
CC	Endoplasmic reticulum lumen (GO:0005788)	49/270	5.10 × 10^−9^
	Focal adhesion (GO:0005925)	58/356	5.10 × 10^−9^
	Secretory granule lumen (GO:0034774)	53/317	9.09 × 10^−9^
	Perinuclear region of cytoplasm (GO:0048471)	53/378	3.82 × 10^−6^
	Cytoplasmic vesicle lumen (GO:0060205)	26/129	8.41 × 10^−6^
MF	Protein homodimerization activity (GO:0042803)	88/664	2.90 × 10^−8^
	Metal ion binding (GO:0046872)	65/442	7.99 × 10^−8^
	Transition metal ion binding (GO:0046914)	56/399	5.30 × 10^−6^
	Kinase binding (GO:0019900)	56/418	2.01 × 10^−5^
	Protein kinase binding (GO:0019901)	60/495	1.73 × 10^−4^
KEGG	Cell cycle	31/124	7.94 × 10^−9^
	Pathways in cancer	72/530	1.08 × 10^−7^
	Complement and coagulation cascades	22/79	2.23 × 10^−7^
	Focal adhesion	37/199	2.27 × 10^−7^
	Human T-cell leukemia virus 1 infection	39/219	2.46 × 10^−7^
WikiPathways	VEGFA-VEGFR2 Signaling Pathway WP3888	48/236	1.93 × 10^−10^
	Cell Cycle WP179	31/120	2.20 × 10^−9^
	Retinoblastoma Gene in Cancer WP2446	25/87	1.20 × 10^−8^
	Nuclear Receptors Meta-Pathway WP2882	50/31	2.81 × 10^−7^
	Human Complement System WP2806	24/97	4.75 × 10^−7^

**Table 7:** Biological functions of 20 hub genes. The biological roles of the 20 core genes are listed.

Gene Symbol	Full Name	Function
MCM2	Minichromosome maintenance complex component 2	Involved in the initiation of eukaryotic genome replication
TOP2A	Topoisomerase IIα	Controls the topology structure of DNA and cell cycle progression
CDC20	Cell division cycle 20	Regulatory protein interacting with several other proteins at multiple points in the cell cycle
CCNB2	Cyclin B2	Essential component of the cell cycle regulatory machinery
BUB1	BUB1 mitotic checkpoint serine/threonine kinase	Serine/threonine-protein kinase that plays a central role in mitosis
TPX2	TPX2 microtubule nucleation factor	Microtubule-associated protein linked to mitosis and spindle assembly
UBE2C	Ubiquitin-conjugating enzyme E2 C	Involved in ubiquitination; required for the destruction of mitotic cyclins and cell cycle progression
CENPF	Centromere protein F	Role in the centromere-kinetochore complex and chromosomal segregation
CDC6	Cell division cycle 6	Essential for the initiation of DNA replication
AURKA	Aurora kinase A	Cell cycle-regulated kinase involved in microtubule formation and/or stabilization at the spindle pole during chromosome segregation
MCM4	Minichromosome maintenance complex component 4	Essential for the initiation of eukaryotic genome replication
TYMS	Thymidylate synthetase	Catalyzes the methylation of deoxyuridylate to deoxythymidylate and maintains the dTMP pool critical for DNA replication and repair
CDKN3	Cyclin-dependent kinase inhibitor 3	Cyclin-dependent kinase inhibitor
PTTG1	PTTG1 regulator of sister chromatid separation	Homolog of yeast securin proteins, which prevent separins from promoting sister chromatid separation
KIF11	Kinesin family member 11	Motor protein that belongs to the kinesin-like protein family
MELK	Maternal embryonic leucine zipper kinase	Serine/threonine-protein kinase involved in various processes such as cell cycle regulation, self-renewal of stem cells, apoptosis, and splicing regulation
FOXM1	Forkhead box M1	Transcriptional activator involved in cell proliferation
HMMR	Hyaluronan (HA) -mediated motility receptor	Binds native and fragmented HA, promotes its uptake
CHEK1	Checkpoint kinase 1	Required for checkpoint mediated cell cycle arrest in response to DNA damage or the presence of unreplicated DNA
TRIP13	Thyroid hormone receptor interactor 13	Interacts with thyroid hormone receptors

**Table 8:** Gene ontology and pathway enrichment analysis of the hub genes.

Category	Term	Count	Adj. *p*-Value
BP	Mitotic cell cycle phase transition (GO:0044772)	9/221	7.22 × 10^−11^
	Regulation of mitotic cell cycle phase transition (GO:1901990)	7/184	3.99 × 10^−8^
	G1/S transition of the mitotic cell cycle (GO:0000082)	5/105	4.03 × 10^−6^
	Regulation of mitotic sister chromatid separation (GO:0010965)	3/15	1.65 × 10^−5^
	DNA metabolic process (GO:0006259)	6/314	1.65 × 10^−5^
CC	Microtubule cytoskeleton (GO:0015630)	8/388	7.67 × 10^−8^
	Spindle (GO:0005819)	6/186	4.15 × 10^−7^
	Spindle pole (GO:0000922)	5/107	7.73 × 10^−7^
	Condensed nuclear chromosome, centromeric region (GO:0000780)	2/12	6.23 × 10^−5^
	Mitotic spindle (GO:0072686)	3/84	6.19 × 10^−4^
MF	Histone serine kinase activity (GO:0035174)	2/7	0.0011
	Histone kinase activity (GO:0035173)	2/9	0.0011
	Kinase binding (GO:0019900)	5/418	0.0011
	Protein kinase binding (GO:0019901)	5/495	0.0018
	Protein serine/threonine kinase activity (GO:0004674)	4/368	0.0059
KEGG	Cell cycle	8/124	2.88 × 10^−12^
	Oocyte meiosis	5/125	8.86 × 10^−7^
	Human T-cell leukemia virus 1 infection	4/219	2.76 × 10^−4^
	Progesterone-mediated oocyte maturation	3/99	4.42 × 10^−4^
	Cellular senescence	3/160	0.0014
WikiPathways	Cell Cycle WP179	8/120	3.37 × 10^−12^
	Gastric Cancer Network 1 WP2361	6/29	3.37 × 10^−12^
	Retinoblastoma Gene in Cancer WP2446	5/87	2.25 × 10^−7^
	Regulation of sister chromatid separation at the metaphase-anaphase transition WP4240	3/15	3.19 × 10^−6^
	DNA Replication WP466	3/42	6.32 × 10^−5^
